# Circulating cell-free DNA content as blood based biomarker in endometrial cancer

**DOI:** 10.18632/oncotarget.23247

**Published:** 2017-12-14

**Authors:** Lucia Cicchillitti, Giacomo Corrado, Martina De Angeli, Emanuela Mancini, Ermelinda Baiocco, Lodovico Patrizi, Ashanti Zampa, Roberta Merola, Aline Martayan, Laura Conti, Giulia Piaggio, Enrico Vizza

**Affiliations:** ^1^ Department of Experimental Clinical Oncology, Gynecologic Oncology Unit, IRCCS Regina Elena National Cancer Institute, Rome, Italy; ^2^ Department of Obstetrics and Gynecology, Gynecologic Oncology Unit, Catholic University of Sacred Heart, Rome, Italy; ^3^ Department of Biomedicine and Prevention, Obstetrics and Gynecology Unit, University of Rome “Tor Vergata”, Rome, Italy; ^4^ Clinical Pathology, IRCCS Regina Elena National Cancer Institute, Rome, Italy; ^5^ Department of Research, Advanced Diagnostics and Technological Innovation, Area of Translational Research, IRCCS Regina Elena National Cancer Institute, Rome, Italy

**Keywords:** endometrial cancer, circulating cell-free DNA, circulating cell-free mitochondrial DNA, bio-markers, liquid biopsy

## Abstract

**Background:**

Altered circulating cell-free DNA (cfDNA) levels are related to cancer development and aggressiveness. Up to now, very few studies have been performed for evaluating cfDNA content in endometrial cancer (EC).

**Methods:**

First, we measured cfDNA release in blood serum of EC cancer patients collected before surgery and before the beginning of any treatment by SYBR Gold assay and correlated it with tumor aggressiveness. We also assessed the relative mitochondrial cell-free DNA (cfmtDNA) content by qRT-PCR. Next, we correlated cfDNA levels with BMI, age, hypertension and inflammation markers.

**Results:**

CfDNA levels are higher in G2 and G3 compared with G1 EC sera. A significant modulation of cfDNA content was detected in sera from patients with BMI>30 compared with those with BMI<30. We observed a further and significant alteration in cfDNA level in hypertensive patients with G2-G3, but not in G1 EC. Analysis of preoperative neutrophil-to-lymphocyte (NLR) and monocyte-to-lymphocyte (MLR) ratios suggests a contribution of the host response in the altered cfDNA levels in EC.

**Conclusions:**

Our data indicate that assessment of total and mitochondrial cfDNA levels in blood sera and the relative NLR and MLR in blood obtained from preoperative patients may help clinical management and prognosis in EC.

## INTRODUCTION

Circulating cell free DNA (cfDNA) are double-stranded molecules with low molecular weight, in the form of short fragments (between 70 and 200 base pairs in length) and/or long fragments up to 21 kb. The presence of cfDNA within the plasma was first reported by Mandel and Metais in 1948 in the blood of healthy individuals [1]. Several decades later, elevated cell-free DNA levels were discovered in the circulation of patients with cancer [2]. CfDNA is released into circulation by various physiologic and pathologic mechanisms. Under normal physiologic circumstances, apoptotic and necrotic cells are cleared by infiltrating phagocytes and cfDNA levels are relatively low. Nevertheless, not all cfDNA originates from cell death. Live cells spontaneously release newly synthesized DNA as part of a homeostatically regulated system [3].

Cancer patients generally have much higher levels of cfDNA than healthy individuals, but the levels vary widely, from 0.01% to more than 90%. The variability of cfDNA levels in cancer patients likely associates with tumor burden, stage, vascularity, cellular turnover, and response to therapy [4].

CfDNA has the advantage to serve as “liquid biopsy”, which may be used as cost-effective and non-invasive surrogates for tissue biopsies in cancer risk prediction and early detection [5]. Moreover, analysis of tumor-specific alterations in cfDNA may represent a tool for cancer detection and disease progression monitoring [6]. However, more studies are needed before liquid biopsies will be routinely used in the clinic.

Recent studies have demonstrated a potential link between circulating cell-free mtDNA (cfmtDNA) content and cancers [7–9]. In particular, evaluation of aberrant changes and altered content of cfmtDNA represents an important approach for early cancer diagnosis with some unique advantages over circulating nuclear cell free DNA (cfnDNA), such as the much shorter and more simply organized mitochondrial genome and the higher number of mtDNA copy. These characteristics make screening much easier and more cost-effective than for the nuclear genome in body fluid samples such as plasma and serum, in which the total DNA concentration is at very low concentration.

Mitochondria are membrane-enclosed organelles in cytoplasm of the eukaryotic cells that play an important role in essential cellular functions such as energy metabolism, synthesis of lipids, amino acids, free radical generation, calcium homeostasis, and apoptosis [10]. Each cell contains several hundred to thousand mitochondria and each mitochondrion possesses its own genome, the mitochondrial DNA (mtDNA), consisting of an approximately 16.5 kb double stranded circular extrachromosomal DNA, maternally inherited. Several reports showed that mitochondrial components and mtDNA, when released into the cytosol and extracellular environment, can directly trigger the innate immune system response [11]. Unlike nDNAs, mtDNA does not have protective histones and sophisticated DNA repair mechanisms, which render it extremely susceptible to oxidative stress and other genotoxic insults [12]. As a defense mechanism to oxidative injury, mitochondria increase their DNA copy numbers to counteract the negative effects resulting from the high rates of mutations in their genomes. The abnormal alteration of mtDNA copy number is well documented for numerous malignancies [13–15], though direct causal links and mechanisms have not yet been established. A recent molecular epidemiological study focused on evaluating the association between mtDNA copy number in peripheral blood leukocytes (PBLs), and EC risk demonstrated that low mtDNA copy number is significantly associated with a more than fivefold increased risk of endometrial cancer compared to those with high mtDNA copy number [16].

EC is the eighth most common type of cancer in women accounting for about 4.8% of all cancers in women (2.3% overall). Several factors have been established to be related with an increased EC risk, such as metabolic syndrome and metabolic abnormality, including hypertension, obesity and diabetes history, low physical activity, and use of unopposed hormone replacement therapy. Up to now few reports have examined cfDNA of EC. A previous study reported that presence of p53 positivity and cfDNA in blood could be used in diagnosis as well as in treatment of Type II EC, thus offering a possibility to individualize treatment regimen [17]. In contrast with these data, Tanaka et al. observed that measurement of cfDNA is not useful for EC screening, but suggested that changes in cfDNA levels in a given patient after surgery/drug treatment may be a prognostic biomarker [18]. Further studies are necessary to understand the role of cfDNA levels as candidate not invasive biomarker in EC. Of note, as yet there is no reported study evaluating cfmtDNA content as complementary tool for EC stratification.

In our study we analysed the total cfDNA and the relative cfmtDNA content in EC blood sera and correlated it with clinical characteristics of patients. Our results provide evidences for association of cfDNA release with EC grading, hypertension and inflammation markers suggesting its role as predictive biomarker in EC.

## RESULTS

### Total cfDNA levels are associated with EC grading

**Figure 1:**
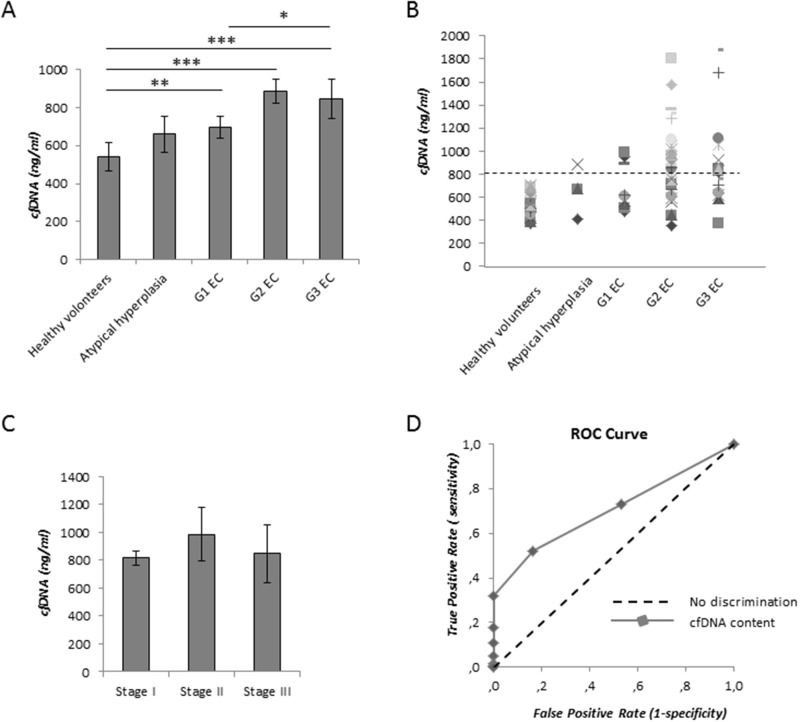
Measurement of cfDNA content with SYBR gold assay in EC patients **(A)** Comparison among cfDNA levesl in serum samples of healthy volunteers (n=21), of patients with atypical hyperplasia (n=6), of G1 EC patients (n=12), G2 EC (n=28), G3 EC (n=17). **(B)** CfDNA distribution in our cohort of serum samples. Dashed line indicates the cutoff value for cfDNA (800 ng/ml). **(C)** Average of cfDNA release in serum samples according to EC stage. Statistical significance: ^*^P≤0.05. Data are represented as mean +/− standard error (SEM). The error bars indicate the standard error. **(D)** Receiver operative characteristics (ROC) curves for cfDNA levels measured with SYBR gold stain resulting by comparing cfDNA levels from healthy volunteers and EC patients.

Table 1 depicts clinical-pathological characteristics of patients enrolled in this study. The blood of cancer patients was obtained before surgery and before the beginning of any treatment. Information about patients was obtained by reviewing their medical charts. Total cfDNA amount was quantified by the direct SYBR gold assay in serum samples. A standard curve obtained with commercial Salmon Sperm DNA was used to extrapolate DNA concentrations. The fit of the standard curve (*R^2^*) was higher than 0,98. Augmented concentrations of cfDNA levels were observed in patients with atypical hyperplasia and a significant rice was detected in EC compared with healthy and noncancer control group (Figure 1A). In addition, a further and significant increase of cfDNA was observed in G2 and G3 (*P*<0.05) compared with G1 EC samples (Figure 1A and Table 2). The distribution of cfDNA values are represented in Figure 1B.

**Table 1:** Clinico-pathological features of our cohort of 59 EC patients

Endometrial cancer (grade)	G1	G2	G3
*No of cases*	12	30	17
*Median Age (years)*	57 (range 48-71)	57 (range 30-73)	61 (range (52-78)
*<65 years old (%)*	58.3	76.6	64.7
*>65 years old (%)*	41.7	23.4	35.3
*BMI <30 (%)*	58.3	53.3	70.5
*BMI >30 (%)*	41.7	46.7	29.5
*Figo Stage (%)*			
*I*	100	89.6	41.2
*II*	0	6.8	23.5
*III*	0	3.4	35.3
*IV*	0	0	0
*Hypertensive (%)*	50	32	58.8
*MI<50%*	100	68.9	11.7
*MI >50%*	0	31.1	88.3
*LSVI (%)*	0	10	70.5
*Lymph node metastases (%)*	0	0	17.6

**Table 2:** Cluster analysis of EC serum samples

	Total cfDNA concentration (ng/ml) ±SD
G1 EC	645.6 ± 253.5
G2 EC	886.1 ± 336.7
G3 EC	846.3 ± 424.6
Non-hypertensive G1 EC	661.7 ± 224.7
Hypertensive G1 EC	668.9 ±324.8
Non-hypertensive G2 EC	711.2 ±211.9
Hypertensive G2 EC	912.0 ±216.2
Non-hypertensive G3 EC	622.9 ±298.5
Hypertensive G3 EC	897.1 ±396.2

Mean of cfDNA concentration ±SD in G1, G2 and G3 EC serum samples and in the relative subgroups of hypertensive and non- hypertensive G1, G2 and G3 EC patients. SD: standard deviation.

**Figure 2:**
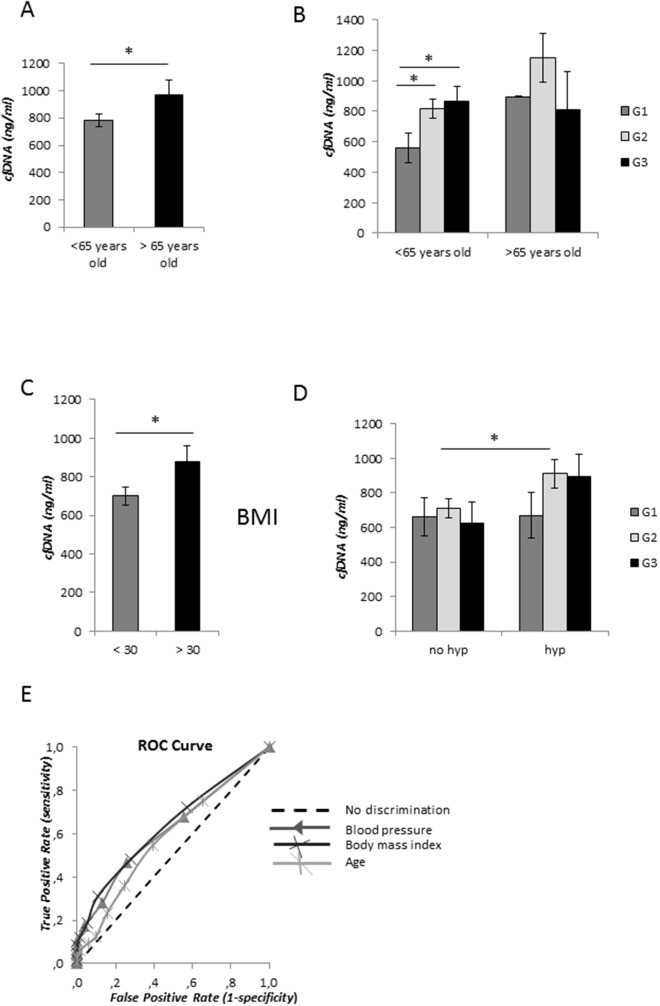
cfDNA concentration levels and metabolic syndrome components **(A)** Average of cfDNA content in patients younger and older than 65 years old. **(B)** Correlation of age with EC grading. **(C)** CfDNA content association with body mass index (BMI) <30 and > 30. **(D)** Correlation analysis of cfDNA amount in serum samples from non-hypertensive (no hyp) and hypertensive (hyp) EC patients. Statistical significance: ^*^P≤0.05, ^**^P≤0.01, ^***^P≤0.001. Data are represented as mean +/− standard error (SEM). The error bars indicate the standard error. **(E)** Receiver operative characteristics (ROC) curves for cfDNA levels discriminating between hypertensive and non- hypertensive patients, patients with BMI ≥ 30 and with BMI < 30, and aged ≥65 and aged < 65 years.

In order to evaluate the predictive capability (i.e. clinical significance) of cfDNA measurements with SYBR gold stain, we performed the receiver operating characteristic (ROC) analysis. The ROC curve obtained by plot at different cut-offs is shown in Figure 1D. We identified a cut-off equal to 800 ng/ml that maximizes sensitivity and specificity that best discriminated cfDNA content between healthy women and EC patients (Table 3). According to this cut-off, we clustered samples with cfDNA levels ≥800 ng/ml and <800ng/ml. At this selected value, we observed that 33.3%, 56.6% and 52.9% of cases displayed high cfDNA amount (≥800 ng/ml) in serum sample from G1, G2 and G3 EC patients, respectively. Through this analysis we did not find any association between cfDNA levels and EC stage (Figure 1C), thus suggesting that high cfDNA levels are strictly associated with EC grading (Figure 1B and Table 2). We then correlated total cfDNA content with ageing. We observed an increased level of cfDNA in sera from EC patients older than 65 years old compared with younger patients (*P*<0.05) (Figure 2A). This increase was not related to EC grading in older patients (Figure 2B). Conversely, in younger patients cfDNA release was directly related with grading, being significantly larger in patients with higher grade EC compared with G1 EC (*P*<0.05) (Figure 2B).

**Table 3:** Receiver operative characteristics (ROC) analyses

Receiver operative characteristics analysis	EC patients *vs.* healthy volunteers	Hypertensive *vs.* non-hypertensive EC patients	Body mass index < 30 *vs.* ≥ 30	Age < 65 years *Vs.* ≥ 65 years
area under the ROC curve (95% confidence interval)	0,704 (0.632 to 0.777)	0.618 (0.521-0.714)	0.634 (0.538-0.739)	0.584 (0.496-0,671)
Sensitivity at the best cut-off (cfDNA=800 ng/ml)	52.1%	46,7%	48,2%	54,7%
Specificity at the best cut-off (cfDNA=800 ng/ml)	83.9%	74,1%	72,2%	60,6%

Receiver operative characteristics (ROC) results and optimal cut-offs values for cfDNA in G1, G2 and G3 EC sera versus those from healthy volunteers, in samples from hypertensive versus non-hypertensive EC, in those from patients with BMI ≥ 30 versus BMI < 30, and aged ≥65 versus aged < 65 years.

### Total cfDNA levels are augmented in hypertensive patients with high grade EC

We focused our study on evaluation of the possible contribution of body mass index (BMI) and high blood pressure in EC serum cfDNA altered levels. We detected a significant increase of total cfDNA amount in sera from EC patients with BMI≥30 compared with those with BMI<30 (*P*<0.05) (Figure 2C), however this elevated level was not associated with the relative EC grade or stage. Very similar amount of cfDNA was observed in serum samples from non-hypertensive patients in G1, G2 and G3 ECs. We observed a significant increase of cfDNA release in hypertensive compared with non-hypertensive patients in sera collected from G2-G3 (*P*< 0.05), but not in those derived from G1 EC (Figure 2D and Table 2), thus suggesting a correlation between elevated levels of total blood serum cfDNA (> 800 ng/ml) and elevated high blood pressure in more aggressive EC.

According to these data, to better investigate the correlation between hypertension and EC aggressiveness we employed the logistic regression analysis. The model predicted a significant and positive correlation between hypertension and higher EC grading (P< 0.02), with 80% decreased risk to have hypertension for G2 EC patients compared with those with G3 EC (Odds ratio [OR] = 0.196, 95% confidence interval [CI] = 0.050-0.761).

### Relationship between cfDNA content and with risk factors

We performed the ROC analysis for different cfDNA values in EC patients older than 65 years old versus younger than 65 years old, hypertensive versus non-hypertensive patients, and patients with BMI > 30 versus BMI ≥30. The ROC curves obtained by plot at different cut-offs of cfDNA concentrations are shown in Figure 2E and analysis described in Table 3. All the three markers showed a low predictive accuracy.

Binary logistic regression analysis was also conducted with reporting of odds ratio to establish the association of cfDNA content ≥ 800 ng/ml with hypertension, ageing and overweight (Table 4). From the model, cfDNA values ≥ 800 ng/ml positively correlate with high blood pressure (p < 0.05), BMI ≥ 30 (p < 0.05), and age ≥ 65 years (p < 0.02).

**Table 4:** Binary logistic regression analysis

Variable	SE	OR	OR (95% CI)	Wald Chi-Square	p-value
Hypertensive	0.609	3.161	0.958-10.432	35.698	0.05
Overweight	0.664	4.664	1.269-17.139	53.780	0.02
Ageing	12.720	21.189	1.752-256.630	53.630	0.02

Logit model applied to examine the association of cfDNA content ≥ 800 ng/ml with hypertension (hypertensive *versus* non-hypertensive), ageing (aged≥ 65 years *versus* < 65 years), and overweight (BMI≥30 *versus* BMI<30).

SE: standardization error; OR: odds ratio; CI: confidence interval.

### Relative cfmtDNA content is inversely related with total cfDNA levels

To better characterize the involvement of cfDNA in EC, we assessed the cfmtDNA content in our cohort of EC blood serum samples. Purified serum cfDNA was isolated and both the cfmtDNA and cfnDNA amount were measured by quantitative real-time polymerase chain reaction (qRT-PCR) followed by evaluation of cfmtDNA content relative to cfnDNA calculated as 2 × 2(nDNA CT – mtDNA CT) [16, 17]. Two primer pairs were used for the amplification of two mitochondrial genes (*MT-ND1* and *mtDNA 16S*) and for the amplification of single-copy nuclear genes (*HGB* and *36B4*). Similar ct values were obtained for both the two mitochondrial and the two nuclear amplicones. The relative cfDNA content was calculated using the average of each qRT-PCR reaction.

**Figure 3:**
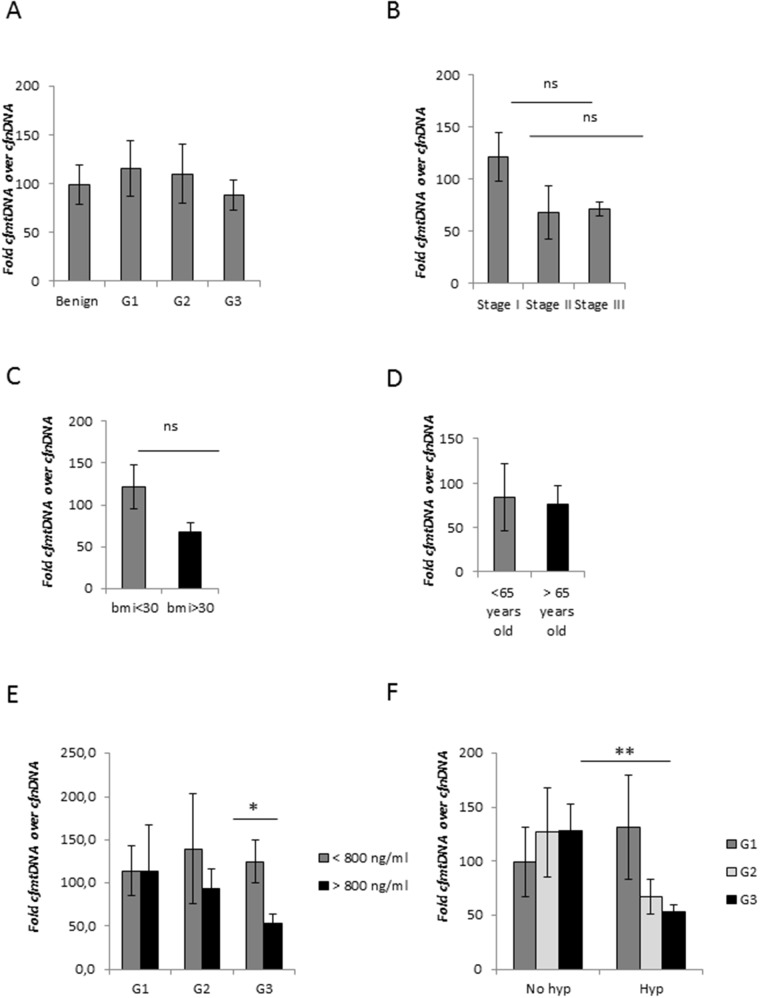
CfmtDNA analysis in EC patients **(A)** Comparison between average of the relative cfmtDNA content in healthy volunteers and patients with EC (G1, G2 and G3), and **(B)** in different EC stage. **(C)** Average of the relative cfmtDNA amount in EC patients with BMI <30 and > 30, and **(D)** younger and older than 65 years old. **(E)** Correlation analysis between the relative cfmtDNA content with cfDNA concentration ≥800 ng/ml or < 800 ng/ml. **(F)** Evaluation of the relative cfmtDNA release in amount in serum samples from non-hypertensive (no hyp) and hypertensive (hyp) patients with G1, G2 and G3 EC. Statistical significance: ns=not significant, ^*^P≤0.05, ^**^P≤0.01. Data are represented as mean +/− standard error (SEM). The error bars indicate the standard error.

We did not detect any alteration in the relative cfmtDNA content in EC compared to noncancer group and also among cancer grade and stage (Figure 3A and 3B). ROC analysis confirmed the poor predictive capability of the relative cfmtDNA content to discriminate between healthy group and EC patients, with AUC equal to 0.51. However, we observed a trend towards lower relative cfmtDNA levels in patients with BMI≥30 (Figure 3C), inversely related with total cfDNA content (Figure 2C). No alterations were detected related to age (Figure 3D). Clustering samples with total cfDNA content >800 ng/ml and ≥800 ng/ml, we observed a lower relative cfmtDNA content in serum samples with higher cfDNA levels only in G2-G3 EC (Figure 3E). A further and significant decrease was observed in G3 EC blood sera (P<0.05). According to the mean of the cfmtDNA relative content measured in subgroups of G1 sera (Figure 3E), we decided to cluster samples with cfmtDNA content <100 and ≥100 in order to evaluate its correlation with EC grade. This analysis showed an increased percentage of samples with relative cfmtDNA content <100 in higher grade EC (Table 5). The relative cfmtDNA amount in hypertensive and non-hypertensive EC patients was also analysed. In non-hypertensive patients, cfmtDNA levels were independent on the EC grade, while a decreased relative cfmtDNA content was associated with hypertension only in blood sera from G2-G3 EC patients, but not with G1 E (Figure 3F), inversely correlated with data obtained with total cfDNA (Figure 2D). This correlation was statistically significant in G3 EC samples (p<0.005) (Figure 3F).

**Table 5:** Total (cfDNA) and relative mitochondrial cfDNA (cfmtDNA) content in our cohort of EC patients

Endometrial cancer (grade)	G1	G2	G3
*Total cfDNA ng/ml (Average concentration) ± SD*	645.6 ± 253.5	886.1 ± 336.7	846.3± 424.6
*Relative cfmtDNA (Average concentration) ± SD*	116.0 ± 28.2	121.0 ± 33.8	93.4 ± 15.7
*Total cfDNA ≥800 ng/ml (%)*	33.3	56.6	52.9
*Relative cfmtDNA <100 (%)*	44.4	68.9	72.2
*Hypertensive with total cfDNA ≥ 800 ng/ml (%)*	50.0	71.4	70.0
*Non-hypertensive with total cfDNA > 800 ng/ml (%)*	25	35.7	16.6
*Hypertensive with relative cfmtDNA <100 (%)*	25.0	71.4	100
*Non-hypertensive with relative mtcfDNA <100 (%)*	50	73.6	28.5

Cluster analysis of cfDNA content (total cfDNA and relative cfmtDNA) in G1, G2 and G3 EC sera and blood pressure level (hypertensive or non-hypertensive). SD: standard deviation.

### Correlation between host response and cfDNA in EC

**Figure 4:**
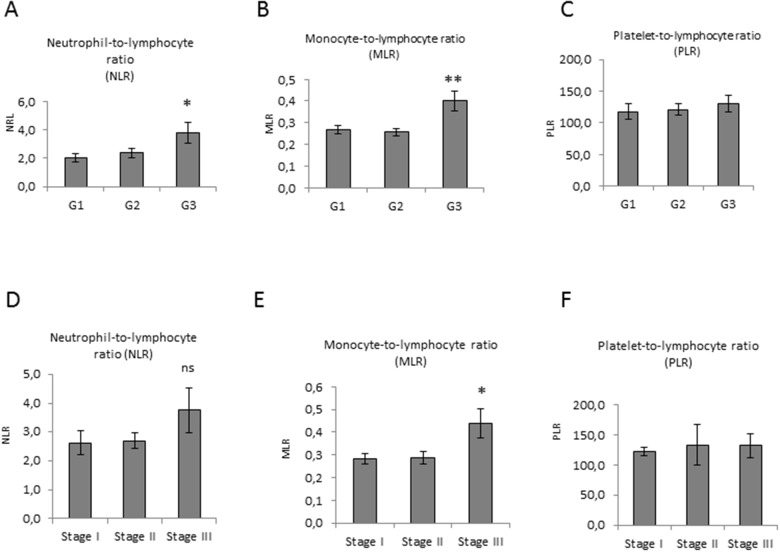
Pre-operative inflammation markers level in EC **(A**, **B**, and **C)** Correlation analysis of NLR, MLR and PLR ratios and EC grade, and **(D**, **E**, and **F)** stage. Statistical significance: ns=not significant, ^*^P≤0.05, ^**^P≤0.01. Data are represented as mean +/− standard error (SEM). The error bars indicate the standard error.

**Figure 5:**
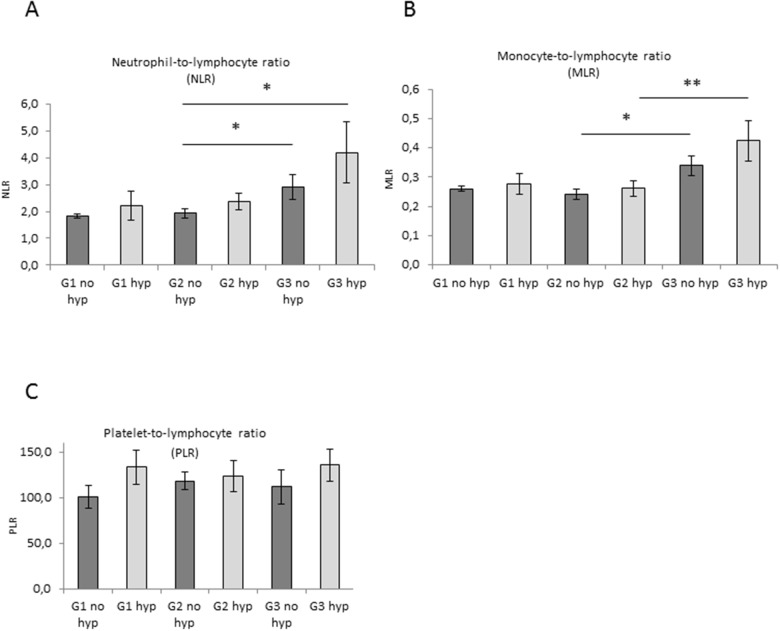
Correlation between pre-operative inflammation markers level and blood pressure in EC **(A**, **B**, and **C)** Evaluation of NLR, MLR and PLR ratio in serum samples from non-hypertensive (no hyp) and hypertensive (hyp) patients with G1, G2 and G3 EC. Statistical significance: ^*^P≤0.05, ^**^P≤0.01. Data are represented as mean +/− standard error (SEM). The error bars indicate the standard error.

To correlate data of cfDNA levels with variations in systemic inflammatory response, we analysed the neutrophil-to-lymphocyte ratio (NLR), the monocyte-to-lymphocyte ratio (MLR) and the platelet-to-lymphocyte ratio (PLR) in our cohort of patients (Supplementary Table 1). A significant rise in NLR and MLR was observed in G3 EC compared with G1 and G2 (Figure 4A and 4B). Higher NLR and MLR were detected in stage III (Figure 4D and 4E). In G3 EC patients with high blood pressure a significant increase of both NLR and MLR was detected (Figure 5A and 5B). These data indicate a correlation between NLR and MLR and cfDNA altered levels in EC. Results obtained on PLR did not show any modulation (Figure 4C, 4F and Figure 5C).

## DISCUSSION

EC is the most frequent tumor in the female reproductive system. EC is classified into two distinct groups, type I and type II, based on histology, which differs in molecular, clinical and histopathological characterisctics. However, treatment of the diversity of this cancer presenting in the clinic is not sufficiently personalized at present.

In this study, we analyzed cfDNA level in serum from EC patients to correlate its content with EC subtypes. All methods for accurate measurement of serum cfDNA are work-intensive and expensive requiring DNA extraction, gel electrophoresis and RT-PCR amplification with specific primers. Consequently, cfDNA measurements are not used during routine patient management. Goldshtein et al. developed an assay based on use of the fluorochrome SYBR Gold stain, which does not require prior processing of samples and is a simple, inexpensive and accurate test for total cfDNA quantification [21]. In the paper using the SYBR Gold assay the authors demonstrated that identical results were obtained in plasma and serum, and showed its specificity and its significant correlation in terms of linearity with conventional qPCR. In our study, we also confirmed that very comparable cfDNA levels are present in serum and plasma samples (data not shown). However, methods including DNA extraction steps and qPCR very likely compromise the yield due to the possible DNA lost during processing and the lack of amplification of small nucleid acids fragments, thus the SYBR Gold stain may represent a better tool in terms of absolute cfDNA quantification since it does not require prior processing of samples and further amplification steps. Several studies have already shown the specificity and clinical relevance of this fluorometric assay [22–26]. In our analysis, total cfDNA concentration range in healthy female volunteers was very similar to that determined in a previous analysis performed using serum samples from 45 women [24], indicating the reproducibility and accuracy of this method.

CfDNA includes also cfmtDNA and cfmtDNA copy number alteration in the peripheral blood has been correlated to inflammatory and pathological processes, where the loss of cell membrane integrity leads to the release of intracellular content [13–15]. However, up to the best of our knowledge, there is no reported study evaluating the association between cfmtDNA content and the risk of development of EC.

In this study, we detected augmented levels of total cfDNA in serum of EC compared with those with benign lesions and observed that this increase was significantly larger in high grade EC. Also, a significant modulation of both total and cfmtDNA content was detected in blood sera derived from the subgroup of hypertensive compared with the set of non-hypertensive high grade EC patients. The same behavior was not observed in the cohort of G1 EC patients. A recent systematic review and meta-analysis showed that a history of hypertension is a factor predisposing to EC reporting that hypertensive women may have a 61% increased of risk of developing this tumor [27]. However, in the mentioned study the authors were not able to identify the association between hypertension and specific EC subtypes. The biological mechanism(s) that may explain the adverse effect of increased levels of blood pressure on EC risk are unclear at present. Hypertension is also associated with dysfunction of the vascular endothelium, that is a hallmark of human diseases characterized by a shift in the actions of the endothelium toward reduced vasodilation, a proinflammatory state, and prothrombic properties. Several papers suggested that cfDNA release is strictly dependent on the level of inflammation and in disease states where cfDNA increases, the clearance capacity of phagocytic cells may be overwhelmed [28, 29].

The cancer incidence at ages >65 years is ∼10 times higher compared with that in the younger population, and it has been shown that approximately 50% of all EC cases are diagnosed in patients aged >65 years [30]. Moreover, EC patients older than 65 years are significantly more likely to develop serous or clear-cell histology and high-grade tumors compared with the younger group [31]. According to these evidences, we decided to cluster samples for patients aged< 65 and aged ≥65 years. We observed a significant increase of cfDNA level in patients older than 65 years old compared to younger EC patients, in accordance with previous evidences [32, 33]. Only in younger patients (<65 years old) we detected an augmented release of cfDNA in higher grade EC. Based on these evidences, we hypothesize that in low grade EC patients the cell death toll may be still not high and very likely under the threshold of the clearance capacity of phagocytic cells, leading to the release of lower amounts of cfDNA and resulting in a similar cfDNA content in hypertensive and non-hypertensive patients.

Tumor growth and development occur as a consequence of interactions between cancer cells and the host immune system. Altered levels of NLR, MLR and PLR have been demonstrated to yield important clues as predictive values in the prognosis and follow-up in several cancer types [34–36] and also in EC [37, 38]. A recent meta-analysis study suggested the prognostic value of NLR in several gynecologic cancers, including EC, and the association of high NLR with worse overall survival (OS) and decreased event-free survival (EFS) [39]. In accordance with these data, we found a significant increase in NLR and MLR in high grade EC. A trend towards higher levels of monocytes and neutrophils was detected in blood of hypertensive EC patients. However, we did not observe any modulation regarding platelet content.

Neutrophils are the most predominant leukocyte subset and they play a crucial role in immunity and systemic and cancer-related inflammation [40]. Recently, several studies have focused on the role of neutrophil extracellular traps (NET) in tumor development including tumor growth, metastasis and angiogenesis [41, 42]. The contribution of NETs to the total cfDNA found in cancer blood remains to be addressed. The mechanism of the release of the chromatin by stimulated neutrophils, called NETosis, may unveil unexpected functions of neutrophils in cancer development and can provide another explanation for the elevated cfDNA release in blood stream in pathologic conditions.

It is possible to speculate that the inflammatory/hypoxic environment of the tumor may trigger neutrophil recruitment thus further increasing total cfDNA content through both NETs deposition on the microvasculature and subsequent entrapment of cancer cells in blood stream, and through NETosis. This mechanism may be very likely dependent on EC grading and aggressiveness. Neutrophils possess a very limited number of mitochondria with exceptional activity, since they lack life-maintaining function, but are involved in regulation of cell death. Level of NETosis activation may be also correlated with the lower relative cfmtDNA content observed in our cohort of serum samples from high grade EC patients.

NETs provide a scaffold for platelet and red blood cell adhesion and aggregation promoting thrombosis and enhancing coagulation [43, 44]. Moreover, neutrophils express and secrete the vast majority of soluble factors involved in tumor progression. Several evidences suggest that cancer cells may manipulate neutrophils, sometimes early in their differentiation process, to create diverse phenotypic and functional polarization states able to alter tumour behavior [40].

Our data strongly suggest that combining both total and mitochondrial cfDNA content, and preoperative NLR and MLR may provide additional tools for EC patient stratification, and may represent important novel biomarker for implementation of clinical management. Moreover, evaluation of neutrophil counts and NETs biomarkers could be used also to evaluate propensity to thrombosis and may serve as prognostic and/or predictive biomarkers in cancer patients.

Further analysis and a prospective clinical trial with robust protocol-driven disease status assessments are needed to validate our observations. Future efforts are necessary to better address the importance of cfDNA test in EC diagnosis and prognosis, in particular focusing on its correlation with patient follow-up and combining results with specific markers of tumor origin [43, 44], and genomic alteration related to EC, as indicated by the Cancer Genome Atlas Research Network (TCGA) [45].

## MATERIALS AND METHODS

### Patient cohort

All healthy volunteers and EC patients were recruited at the Regina Elena National Cancer Institute. We collected serum samples from 22 healthy volunteers and 59 EC patients. According with the histologic grade, we analyzed samples from 12 G1, 30 G2 and 17 G3 ECs. Table 1 depicts clinical-pathological characteristics of patients enrolled in this study. The blood of cancer patients was obtained before surgery and before the beginning of any treatment. Information about patients was obtained by reviewing their medical charts.

The NLR, NLR and PLR were defined as the absolute neutrophil count in peripheral blood divided by the absolute lymphocyte count, the absolute count of monocyte divided by the absolute lymphocyte count, and the absolute platelet count in peripheral blood divided by the absolute lymphocyte count, respectively. The blood tests were obtained within 24 hours for all patients as routine clinical practice before surgery.

### Serum blood samples storage

Whole blood samples were collected in Vacutainer tubes without anticoagulant. Blood was left at room temperature to allow to clot. Serum was separated by centrifugation at 1,000–2,000 x g for 10 minutes in a refrigerated centrifuge and stored at −20°C or lower.

### DNA extraction

Isolation from 1 ml of blood serum was performed with commercial kit for blood DNA according to manufacturer’s specifications from Qiamp Circulating Nucleid Acid kit (QIAGEN).

### Measurement of cfDNA levels

SYBR Gold Nucleic Acid Gel Stain (Invitrogen) was diluted first at 1:1000 in dimethyl sulphoxide (DMSO) and then at 1:8 in phosphate-buffered saline (PBS). Ten microliters of DNA solutions or sera were applied to a black 96-well plates. Forty microliters of diluted SYBR Gold were added to each well (final dilution 1:10,000) and fluorescence measured with a 96well fluorometer at an emission wavelength of 535 nm and an excitation wavelength of 485 nm. Serum samples were diluted in PBS fivefold (20%). Assay was performed in triplicate. Standards were prepared with commercial Salmon sperm DNA.

### Quantitative real-time polymerase chain reaction (qRT-PCR)

Both the mtDNA and nDNA content were measured by qRT-PCR using SYBR Green Master Mix (Applied Biosystems, CA, USA) followed by evaluation of the average CT values from triplicate reactions from Real Time PCR software. Primers were designed and used for relative quantification for mtDNA to nDNA content. Two primer pairs were used for the amplification of two mitochondrial genes: *MT-ND1* and mtDNA 16S. Two primer sets were used for the amplification of the single-copy nuclear gene human globulin (HGB) and 36B4. Primers used are listed in Supplementary Table 2.

Briefly, 10 μL of qRT-PCR reaction for each gene consists of 1 X SYBR Green Master Mix (Applied Biosystems, CA, USA), 200 nmol/L each primer, and 1 μL of purified serum DNA sample diluted 1:6. To determine the mtDNA content relative to nDNA, the following equations were used as previously described [19, 20].

 ΔCT = (nDNA CT – mtDNA CT).

 Relative mitochondrial DNA content = 2 × 2ΔCT

### Statistical analysis

Data were reported as mean and standard deviation. In all experiments, comparisons of results between two groups were based on Student’s t-test and one-way analysis of variance (ANOVA). P≤0.05 was deemed to be significant variation. Receiver operating characteristic (ROC) curves were employed and area under the curve (AUC) calculated to evaluate diagnostic accuracy, classified as low (0.5 < AUC ≤ 0.7), moderate (0.7 < AUC ≤ 0.9) or high (0.9 < AUC ≤ 1.0). Logistic regression model was used to estimate ORs and 95% CIs, and statistical analyses were performed using SAS version 9.2 (SAS Institute, Cary, NC).

### Ethics approval and consent to participate

The experimental protocol was approved by the Ethics Committee of the Regina Elena National Cancer Institute, Rome, Italy (RS: 2021/2017), and performed in accordance with the relevant guidelines and regulations. Written informed consent was obtained from all patients.

## SUPPLEMENTARY MATERIALS TABLES



## Abbreviations

cfDNA: cell-free DNA
cfmtDNA: mitochondrial cell-free DNA
NLR: neutrophil-to-lymphocyte ratio
MLR: and monocyte-to-lymphocyte ratio
PLR: platelet-to-lymphocyte ratio
BMI: body mass index
NET: neutrophil extracellular trap
AUC: area under the curve
OR: odds ratio
CI: confidence interval
