# Supplementary material for: Circulating cell-free DNA content as blood based biomarker in endometrial cancer
Source: Oncotarget. 2017 Dec 14;8(70):115230–43. doi: 10.18632/oncotarget.23247 (PMC5777767; doi:10.18632/oncotarget.23247)
Supplement: Supplementary file 1 [file oncotarget-08-115230-s001.pdf]

Circulating cell-free DNA content as blood based biomarker in endometrial cancer

SUPPLEMENTARY MATERIALS

Supplementary Table 1: NLR, MLR and NLR in our cohort of EC patients. Cluster analysis in G1, G2 and G3 EC and blood pressure level (hypertensive or not hypertensive)

Endometrial cancer (grade)	G1	G2	G3
Platelet-to- lymphocyte ratio (PLR) Average concentration Range	122,7± 15,9 71,4-208,6	122,1± 8,7 51,3-239,7	122,7± 13,3 50,9-220,8
Neutrophil-to-lymphocyte ratio (NLR) Average concentration Range	1,9 ± 0,4 1,1-4,1	2,4 ± 0,3 0,9-8,4	3,8 ± 0,7 1,3-13,5
Monocyte-to-lymphocyte ratio (MLR) Average concentration Range	0,3 ± 0,03 0,2-0,4	0,3 ± 0,02 0,1-0,4	0,4 ± 0,05 0,3-0,9
Not hypertensive with NLR >2,0 x10^3 (%)	40	26,6	71,4
Hypertensive with NLR >2,0 x10^3 (%)	40	55,5	70
Not hypertensive with MLR >0,3 x10^3 (%)	0	13,3	42,8
Hypertensive with MLR >0,3 x10^3 (%)	40	22,2	70

Supplementary Table 2: List of primers for mtcfDNA relative content assessment

FWD ND1	5'-CCCTAAAACCCGCCACATCT-3'
REV ND1	5'-GAGCGATGGTGAGAGCTAAGGT-3'
FWD mtDNA 16S	5'-CAGCCGCTATTAAAGGTTCG-3'
REV mtDNA 16S	5'-CCTGGATTACTCCGGTCTGA-3'
FWD HGB	5′-GTGCACCTGACTCCTGAGGAGA-3′
REV HGB	5′-CCTTGATACCAACCTGCCCAG-3′
FWD 36B4	5'-CAGCAAGTGGGAAGGTGTAATCC-3'
REV 36B4	5'-CCCATTCTATCATCAACGGGTACAA-3'
